# Relative Entropy of Distance Distribution Based Similarity Measure of Nodes in Weighted Graph Data

**DOI:** 10.3390/e24081154

**Published:** 2022-08-19

**Authors:** Shihu Liu, Yingjie Liu, Chunsheng Yang, Li Deng

**Affiliations:** School of Mathematics and Computer Science, Yunnan Minzu University, Kunming 650504, China

**Keywords:** distance distribution, link prediction, relative entropy, similarity measure, weighted graph data

## Abstract

Many similarity measure algorithms of nodes in weighted graph data have been proposed by employing the degree of nodes in recent years. Despite these algorithms obtaining great results, there may be still some limitations. For instance, the strength of nodes is ignored. Aiming at this issue, the relative entropy of the distance distribution based similarity measure of nodes is proposed in this paper. At first, the structural weights of nodes are given by integrating their degree and strength. Next, the distance between any two nodes is calculated with the help of their structural weights and the Euclidean distance formula to further obtain the distance distribution of each node. After that, the probability distribution of nodes is constructed by normalizing their distance distributions. Thus, the relative entropy can be applied to measure the difference between the probability distributions of the top *d* important nodes and all nodes in graph data. Finally, the similarity of two nodes can be measured in terms of this above-mentioned difference calculated by relative entropy. Experimental results demonstrate that the algorithm proposed by considering the strength of node in the relative entropy has great advantages in the most similar node mining and link prediction.

## 1. Introduction

In the real world, numerous complex networks can be abstracted as graph data, such as social networks [1], protein interaction networks [2], traffic networks [3], and e-commerce networks [4]. The graph data can be used to not only portray the weight information of nodes, but also to describe the topological information between nodes. Therefore, the graph data have been given special attention in many fields due to their quantities of valuable information [5,6]. Especially in recent years, many scholars have gradually focused on the similarity measure of nodes in graph data [7,8]. As a necessary tool to determine the similarity between two nodes, the similarity measure plays a vital role in the most similar node mining [9], link prediction [10], cluster analysis [11], and so on [12,13,14].

Up to now, plenty of similarity measure algorithms have been proposed to calculate the similarity of nodes, and these algorithms can be roughly classified into three categories: local similarity indices [15,16], quasi-local similarity indices [17,18], and global similarity indices [19,20]. These three types of indices include some representative algorithms, such as the common neighbor (*CN*) index [15], Adamic–Adar (*AA*) index [16], local random walk (*LRW*) index [17], average commute time (*ACT*) index [19], and so on [18,20]. The *CN* index calculates the similarity between two nodes by counting their common neighbors. In order to distinguish the contribution of different common neighbors, the *AA* index is presented by employing the degree of common neighbors. *LRW* index and *ACT* index are constructed based on the random walk of particles between two nodes.

In recent years, some similarity measures have been also studied from the perspective of information theory. For example, Tan et al. [21] applied the mutual information to graph data and then designed the mutual information (*MI*) index to calculate the similarity of nodes. Inspired by *MI* index, Zhu et al. [22] used the mutual information to weighted graph data and proposed a weighted mutual information model to explore the influence of strong and weak tie effects. However, if these indices based on the mutual information are used to calculate the similarity of nodes, then these nodes of a larger degree will become general similar nodes [23]. Bear in mind that Zhang et al. [24] presented a local relative entropy (*LRE*) index to calculate the similarity of nodes. In the definition of the (*LRE*) index, the relative entropy is used to measure the difference between the degree distributions of the two nodes utilized to further obtain their similarity. Moreover, Zheng et al. [25] utilized the relative entropy to measure the difference between the transition probability distributions of two nodes and then constructed the *RE-model* to calculate the similarity of nodes.

In the measurement results of these mutual information-based indices, the nodes of a larger degree easily become general similar nodes. In the measurement results of these relative entropy-based indices, the situation that many nodes are similar to the nodes of a larger degree can be avoided. However, the *LRE* index utilizes the degree distribution of each node, and the *RE-model* uses the transition probability distributions between two nodes. Therefore, the degree of nodes is merely utilized in the definition of the two indices. Unfortunately, the *LRE* index and *RE-model* do not make full use of the strength of nodes in weighted graph data, which leads to their performance failing to be improved further. In particular, there is a poor performance when the relative entropy-based similarity measures are applied to carry out the link prediction [26].

Generally speaking, the strength of a node represents its ability to collect information, and the degree of a node represents its ability to diffuse information. Thus, if the similarity measure algorithm is constructed by properly integrating the degree and strength of nodes, then its performance may be further enhanced [27]. To our knowledge, however, there are rare studies on how to improve the performance of the similarity measurement by using the degree and strength of nodes. The similarity measure based on the relative entropy is also given little attention for link prediction in weighted graph data.

Based on the above analysis and discussion, the relative entropy of the distance distribution based similarity measure of nodes is proposed in this paper. The distance distribution of each node can be obtained by calculating the Euclidean distance between the structural weights of two nodes, where the structural weight of each node comprehensively considers its degree and strength in weighted graph data. After that, the probability distribution of nodes is constructed by normalizing the elements in their distance distribution. At last, the relative entropy can be applied to measure the difference between the probability distributions of the top *d* important nodes and all nodes in graph data, which ensures that the similarity of nodes can be calculated with the lower time cost. We numerically simulated the proposed algorithm and verified its effectiveness and efficiency in the most similar node mining and link prediction. In this paper, we provide a similarity measure algorithm with the following several contributions in mind.

- The structural weights of nodes are defined by integrating their degree and strength, and then the structural weights-based distance between two nodes can be calculated.
- The difference between the probability distributions of the top *d* important nodes and all nodes in the graph data is measured by using the relative entropy, which can ensure that the similarity of nodes can be calculated with the lower time cost.
- The proposed similarity measure algorithm has a great advantage in mining the most similar nodes and performing the link prediction, compared with the majority of benchmark algorithms.

The remainder of this paper is organized as follows. Some basic knowledge of the weighted graph data and similarity measure are reviewed in Section 2. The relative entropy-based similarity measure algorithm is defined in Section 3. Some experimental materials are introduced in Section 4. Experimental results are demonstrated in Section 5. The conclusion of this paper is drawn in Section 6.

## 2. Preliminaries

In this section, some necessary knowledge is introduced, including the concepts of weighted graph data, the relationship between the node similarity and link prediction, and the definition of relative entropy.

### 2.1. Weighted Graph Data

Formally, the so-called weighted graph data can be expressed as a 3-tuple *G* = (*V*, *E*, *W*), where $V=\{v_{x}|x=1,2,\cdots ,n\}$ represents the set of nodes, $E=\{e_{xy}|x,y=1,2,\cdots ,n\}$ indicates the set of edges, and $W=\{w_{xy}|x,y=1,2,\cdots ,n\}$ denotes the set of weights. It is not difficult to find that the weighted graph data will degenerate to the unweighted form *G* = (*V*, *E*) if $w_{xy}=1$, and x,y=1,2,⋯,n. Moreover, the $e_{xy}$ represents the edge that connects nodes $v_{x}$ and $v_{y}$, and then $w\left(e_{xy}\right)=w_{xy}$ denotes the weight of the edge $e_{xy}$.

Considering two nodes $v_{x},v_{y}\in V$, they are adjacent to each other if they are two end nodes on the edge $e_{xy}\in E$. Let $a_{xy}$ = 1 and $a_{xy}$ = 0 respectively denote that an edge between $v_{x}$ and $v_{y}$ is existent and non-existent. Then, the adjacency matrix of the graph *G* = (*V*, *E*) is defined by $A={\left\{a_{xy}\right\}}_{n\times n}$. For a weighted graph *G* = (*V*, *E*, *W*), its weighted adjacency matrix can be expressed as $A_{w}={\left\{w_{xy}\right\}}_{n\times n}$.

In order to facilitate the understanding of the content of this article, some relevant notations are summarized in Table 1.

### 2.2. Relationship between the Node Similarity and Link Prediction

In the real world, many graph data are incomplete or inaccurate. These graph data are collected from a wide range of information systems and can only reflect a part of the real information. Thus, link prediction technology becomes a significant and useful tool during the analysis of graph data, as its task is to detect and mine the missing information in graph data. Generally speaking, the link prediction technology aims at quantifying the existence likelihood of a candidate edge between two nodes. In related research, this kind of existence likelihood can be measured by using the similarity between two nodes [28,29,30]. Therefore, the similarity measure algorithm of nodes is an efficient and effective method for performing the link prediction.

In the process of performing link prediction, the observed edge set *E* needs to be randomly divided into the training set $E_{t}$ and the probe set $E_{p}$, where $E_{t}\;\cup \;E_{p}=E\;\mathrm{and}\;E_{t}\;\cap \;E_{p}=\emptyset$. The edges in $E_{t}$ are regarded as known information, which is used to calculate the similarity between two nodes. The edges in $E_{p}$ are applied to test the performance of similarity measure algorithms by making a comparison of similarity score with the edges in edge set U−E. The set U−E expresses the set of unknown edges, and *U* denotes the universal set of all possible edges. Thus, the edges in $E_{t}$ and the edges in U−E make up the set of all missing edges(i.e., $E_{t}\;\cup \left(\;U\;-\;E\right)$) in graph data.

From the above analysis, the relationship between the node similarity and link prediction can be briefly described as follows. Given an edge in $E_{t}\;\cup \left(\;U\;-\;E\right)$, it can be assigned a score by using any kind of similarity measure. After that, all edges in $E_{t}\;\cup \left(\;U\;-\;E\right)$ are sorted in decreasing order according to their scores. Finally, the edge with the highest-ranked score is most likely to exist.

### 2.3. Relative Entropy

In information theory, the relative entropy is also called Kullback–Leibler divergence, which is a measure of the distance between two distributions [31,32]. In general, relative entropy can be used to measure the difference between two probability distributions. Considering two different probability distributions *P* and *Q*, their relative entropy can be described in the following form:

(1) $$D_{KL}\left(P∥Q\right)=\sum_{x=1}^{r}P\left(x\right)\cdot log_{2}\frac{P(x)}{Q(x)},$$

where *r* is the number of components in these two probability distributions *P* and *Q*. The greater the relative entropy between the *P* and *Q*, the greater the difference between them, and vice versa.

Note that the relative entropy is asymmetrical, which is $D_{KL}\left(P∥Q\right)\neq D_{KL}\left(Q∥P\right)$. Therefore, this paper redefined the Kullback–Leibler divergence in the process of the calculation of node similarity. In order to make the relative entropy able to satisfy the definition of the distance, the redefined formula is rewritten as

(2) $$RD\left(P∥Q\right)=\frac{D_{KL}\left(P∥Q\right)+D_{KL}\left(Q∥P\right)}{2}.$$

From the above analysis, there is

$$\begin{array}{cc} RD\left(P∥Q\right) & =\left\{\sum_{x=1}^{r}P\left(x\right)\cdot log_{2}\left[P\left(x\right)/Q\left(x\right)\right]+\sum_{x=1}^{r}Q\left(x\right)\cdot log_{2}\left[Q\left(x\right)/P\left(x\right)\right]\right\}/2\\ & =\left\{\sum_{x=1}^{r}\left[P\left(x\right)-Q\left(x\right)\right]\cdot log_{2}\left[P\left(x\right)/Q\left(x\right)\right]\right\}/2\\ & =\left\{\sum_{x=1}^{r}\left[Q\left(x\right)-P\left(x\right)\right]\cdot log_{2}\left[Q\left(x\right)/P\left(x\right)\right]\right\}/2=RD(Q∥P). \end{array}$$

Therefore, RD(P∥Q) is symmetrical. According to the nature of the Kullback–Leibler divergence, we can know that RD(P∥Q) satisfies the definition of distance measure.

## 3. Method

Aiming at the problem of the node similarity in weighted graph data, a similarity measure algorithm is proposed in this section. The proposed algorithm employs relative entropy to measure the difference between the probability distributions of two nodes. The probability distribution of each node is obtained in terms of its distance distribution. The distance distribution of each node is defined by calculating the Euclidean distance between the structural weights of two nodes. The structural weights of nodes can be defined by utilizing their degree and strength information.

### 3.1. Structural Weight Set of Nodes

In weighted graph data, the connections between nodes are varied, and the degree and strength of the different nodes have great variation. Generally, the strength of a node represents its ability to collect information, but the degree of a node denotes its ability to diffuse information. Bearing in mind the specificity of nodes, the structural weight set of nodes is given in this paper. Before giving the structural weight set of nodes, we define the three kinds of structural weights of nodes.

**Definition** **1**  *(Unit weight of nodes)*. *The unit weight of a node is defined as the average value of the weight for all the edges connecting this node. Then considering a node $v_{x}$ in G = <V, E, W>, its calculation expression of unit weight can be defined by*

(3) $$uw\left(v_{x}\right)=\frac{s_{x}}{k_{x}},$$

*where the definition of $s_{x}$ and $k_{x}$ respectively are described in Table 1 for easy reading, and $uw(v_{x})$ represents the unit weight of $v_{x}$. Clearly, $uw(v_{x})$ simply combines its ability to collect and diffuse information.*

**Definition** **2**  *(Degree weight of nodes)*. *The degree weight of a node fully takes into account its ability to diffuse information in the case of suppressing its ability to collect information. Then, considering a node $v_{x}$ in G = <V, E, W>, its calculation expression of degree weight can be defined by*

(4) $$dw\left(v_{x}\right)=\frac{k_{x}^{2}}{\sum_{y=1}^{n}k_{y}}\cdot \frac{1}{s_{x}},$$

*where $dw(v_{x})$ represents the degree weight of $v_{x}$. It is not difficult to find that the $dw(v_{x})$ considers the impact of diffusing information of all nodes in G = <V, E, W>.*

**Definition** **3**  *(Strength weight of nodes)*. *The strength weight of a node fully considers its ability to collect information under suppressing its ability to diffuse information. Given a node $v_{x}$ in G = <V, E, W>, its calculation expression of strength weight can be defined by*

(5) $$sw\left(v_{x}\right)=\frac{s_{x}^{2}}{\sum_{y=1}^{n}s_{y}}\cdot \frac{1}{k_{x}},$$

*where $sw(v_{x})$ denotes the strength weight of $v_{x}$. It is can be found that the $sw(v_{x})$ takes into account the influence for collecting information of all nodes in G = <V, E, W>.*

**Definition** **4**  *(Structural weight set of nodes)*. *Considering the specificity of the degree and strength of nodes, we define the structural weight set of nodes as*

(6) $$SA\left(v_{x}\right)=\left\{uw\left(v_{x}\right),dw\left(v_{x}\right),sw\left(v_{x}\right)\right\}\;\mathrm{for}\;x=1,2,\cdots ,n,$$

*where $SA(v_{x})$ indicates the structural weight set of $v_{x}$, which consists of the unit weight, degree weight, and strength weight of $v_{x}$.*

### 3.2. Distance Distribution of Nodes

As a frequently used distance measure in mathematics, the Euclidean distance is also widely employed in various similarity measure researches. Its advantage is to overcome the correlation interference between variables and eliminate the influence of the dimension of each variable at the same time. Therefore, Euclidean distance is applied to calculate the distance between the structural weights of two nodes, to obtain the distance distribution of each node in this paper.

In this paper, the three kinds of structural weights of nodes are defined. Thus, the distance between two nodes can be calculated by using the formula of Euclidean distance in 3-dimensional space. In the following, we define the distance between two nodes.

**Definition** **5**  *(Distance between two nodes)*. *In this paper, the distance between two nodes can be calculated by using the formula of Euclidean distance in three-dimensional space and their structural weight set. Considering two nodes $v_{x}$,$v_{y}$ in G = <V, E, W>, then the distance between them can be calculated by*

(7) $$d\left(v_{x},v_{y}\right)=\sqrt{{\left(uw\left(v_{x}\right)-uw\left(v_{y}\right)\right)}^{2}+{\left(dw\left(v_{x}\right)-dw\left(v_{y}\right)\right)}^{2}+{\left(sw\left(v_{x}\right)-sw\left(v_{y}\right)\right)}^{2}},$$

*where $d(v_{x},v_{y})$ expresses the Euclidean distance between $v_{x}$ and $v_{y}$. It is not difficult to find that the distance between any two nodes can be calculated in G = <V, E, W>.*

Based on the above discussion, the distance distribution of each node can be obtained. In the following, we define the distance distribution of nodes.

**Definition** **6**  *(Distance distribution of nodes)*. *In this paper, the distance distribution of each node can be defined in terms of the distance between this node and other nodes. Given a node $v_{x}$ in G = <V, E, W>, its distance distribution can be defined as*

(8) $$DD\left(v_{x}\right)=\left\{d\left(v_{x},v_{1}\right),d\left(v_{x},v_{2}\right),\cdots ,d\left(v_{x},v_{n}\right)\right\},$$

*where $DD(v_{x})$ denotes the distance distribution of $v_{x}$, and one can observe that the distance distribution of each node has n components.*

To use the relative entropy to measure the node similarity, the probability distribution of each node needs to be obtained. Thus, the distance between two nodes should be normalized within the range of [0,1]. In view of the similarity between two nodes being inversely proportional to the distance between them, the probability of an edge existing between two nodes can be calculated by using a constant 1 to subtract the normalized distance between them. On these bases, we define the probability distribution of nodes.

**Definition** **7**  *(Probability distribution of nodes)*. *In this paper, the probability distribution of each node can be constructed by utilizing the normalized distance between it and other all nodes. Given a node $v_{x}$ in G = <V, E, W>, its probability distribution can be expressed as*

(9) $$\begin{array}{cc} PD(v_{x}) & =\left\{p\left(v_{x},v_{1}\right),p\left(v_{x},v_{2}\right),\cdots ,p\left(v_{x},v_{n}\right)\right\}\\ \; & =\left\{1-\frac{d(v_{x},v_{1})}{\sum_{y=1}^{n}d(v_{x},v_{y})},1-\frac{d(v_{x},v_{2})}{\sum_{y=1}^{n}d(v_{x},v_{y})},\cdots ,1-\frac{d(v_{x},v_{n})}{\sum_{y=1}^{n}d(v_{x},v_{y})}\right\}, \end{array}$$

*where $PD(v_{x})$ denotes the probability distribution of $v_{x}$, and $p(v_{x},v_{y})$ is the probability of existing an edge between $v_{x}$ and $v_{y}$, y=1,2,⋯,n.*

### 3.3. Design of Algorithm

From the above discussion, the three structural weight sets of nodes are given by using their unit weight, degree weight, and strength weight. Then, the distance distribution of nodes is also obtained by calculating the Euclidean distance between the structural weights of any two nodes. Furthermore, considering that the similarity between any two nodes is inversely proportional to the distance between them, the probability distribution of nodes is constructed by using a constant 1 to subtract the normalized distance between them to better describe the similarity of the two nodes.

It is not difficult to find that Equation (9) includes the probability between $v_{x}$ and all nodes in graph data. Thus, there may be a large number of resource losses during applying the relative entropy to measure the difference between the probability distributions of two nodes. This is because the dimension of the probability distribution of each node increases as the size of the graph data increases. In addition, some non-significant nodes also affect the accuracy of algorithm. In reality, these nodes that have a greater impact on the similarity calculation which may be able to achieve good results with a low computational cost [24]. Considering the specificity of the degree and strength of nodes, this paper selects the probability distribution of any node in the graph data and the top *d* important nodes to construct for the similarity measure according to the unit weight of all nodes.

First, the top *d* important nodes are found by arranging all nodes in descending order according to their unit weights. Next, the set $S=\{{\hat{v}}_{1},{\hat{v}}_{2},\cdots {\hat{v}}_{d}\}$ of the top *d* important nodes is constructed. Then, the probability between the $v_{x}$ and any nodes in *S* is obtained according to the elements in $PD(v_{x})$. Then, the *d*-dimension vector $V\left(v_{x}\right)=\left[p\left(v_{x},{\hat{v}}_{1}\right),p\left(v_{x},{\hat{v}}_{2}\right),\cdots ,p\left(v_{x},{\hat{v}}_{d}\right)\right]$ is further formed. Finally, the *d*-dimensional probability distribution of $v_{x}$ is given by normalizing the elements in $V(v_{x})$, which is

$$\hat{PD}\left(v_{x}\right)=\left[\hat{p}\left(v_{x},{\hat{v}}_{1}\right),\hat{p}\left(v_{x},{\hat{v}}_{2}\right),\cdots ,\hat{p}\left(v_{x},{\hat{v}}_{d}\right)\right],$$

where the $\hat{p}\left(v_{x},{\hat{v}}_{z}\right)=p\left(v_{x},{\hat{v}}_{z}\right)/\sum_{z=1}^{d}p(v_{x},{\hat{v}}_{z})$.

From the above analysis, the difference between the probability distributions of the top *d* important nodes and any node in graph data can be measured by utilizing the relative entropy. In the following, the relative entropy value between the probability distributions of the top *d* important nodes and any node in graph data can be calculated.

**Definition** **8**  *(Relative entropy value between the probability distributions of two nodes)*. *Considering a pair of node ($v_{x}$, $v_{y}$) in G = <V, E, W>, the relative entropy value between their probability distributions can be defined as
*

(10) $$\begin{array}{cc} RE(v_{x},v_{y}) & =D_{KL}\left(\hat{p}\left(v_{x},{\hat{v}}_{z}\right)\left|\right|\hat{p}\left(v_{y},{\hat{v}}_{z}\right)\right)\\ \; & =\sum_{z=1}^{d}\hat{p}\left(v_{x},{\hat{v}}_{z}\right)\cdot log_{2}\frac{\hat{p}\left(v_{x},{\hat{v}}_{z}\right)}{\hat{p}\left(v_{y},{\hat{v}}_{z}\right)}, \end{array}$$

*where $RE(v_{x},v_{y})$ denotes the relative entropy value between the probability distributions of $v_{x}$ and $v_{y}$. In this paper, the $ln\left(\hat{p}\left(v_{x},{\hat{v}}_{z}\right)/\hat{p}\left(v_{y},{\hat{v}}_{z}\right)\right)$ is specified as 0 when $\hat{p}\left(v_{x},{\hat{v}}_{z}\right)$ = 0 or $\hat{p}\left(v_{y},{\hat{v}}_{z}\right)$ = 0.*

**Definition** **9**  *(Difference between two nodes)*. *In this paper, the difference between two nodes is calculated by employing the relative entropy value between them. Considering two nodes $v_{x}$ and $v_{y}$ in G = <V, E, W>, the difference between them can be expressed by*

(11) $$d_{xy}=\frac{RE\left(v_{x},v_{y}\right)+RE\left(v_{y},v_{x}\right)}{2},$$

*where $d_{xy}$ denotes the difference between $v_{x}$ and $v_{x}$. Clearly, the $d_{xy}$ is symmetrical in terms of Equation (2), and then it can be used to calculate the similarity of nodes.*

Generally speaking, the greater the difference between the probability distributions of two events, the smaller their similarity. Thus, the relative entropy of distance distribution based similarity measure of nodes is proposed to transform the difference between two nodes into their similarity.

**Definition** **10**  *(Relative entropy of distance distribution based similarity measure of nodes, **REDD**)*. *In this paper, the similarity of two nodes can be represented by their difference with the help of the similarity measure algorithm**REDD**index. Considering two nodes $v_{x}$ and $v_{y}$ in G = <V, E, W>, the**REDD**index can be expressed as*

(12) $$s_{xy}^{REDD}=1-\frac{d_{xy}}{d_{max}},$$

*
where $d_{max}$ is the maximum of the difference between any two nodes in graph data, $s_{xy}^{REDD}$ is the similarity of $v_{x}$, and $v_{y}$ calculated by the REDD index. REDD is the abbreviation of the algorithm we proposed, and its corresponding pseudo-code is outlined in Algorithm 1.
*

**Algorithm description:** The input is the weighted graph data G = <V,E,W> and dimension *d*, the output is the similarity matrix $S_{n\times n}^{REDD}$. The construction procedure of the *REDD* index is operated in the following three phases: initialization phase (line 2), computation phase (lines 4–12), and update phase (line 14). The initialization phase refers to assigning certain storage to the matrix $S_{n\times n}^{REDD}$. The computation phase iteratively calculates the similarity of two nodes by using the previous definitions. The purpose of the update phase is to store the similarity of all node pairs in the matrix $S_{n\times n}^{REDD}$.
**Algorithm 1** The construction procedure of REDD index.

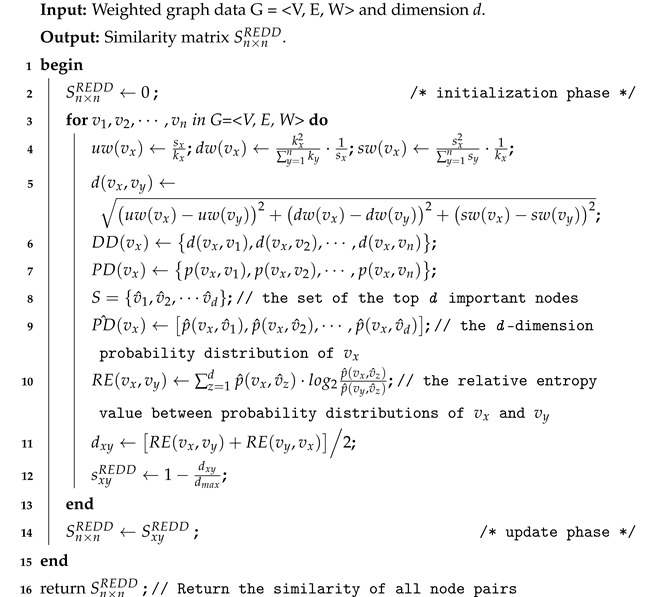



## 4. Materials

In this section, we introduce some experimental materials, such as the experiment datasets, benchmark algorithms, and evaluation metrics. Moreover, the experimental environment used in this paper is listed in Table 2.

### 4.1. Datasets Description

In this article, we consider 12 real-world weighted graph data that are freely downloaded from some public academic websites. These weighted graph data include the transportation network, citation network, ecology network, and biological network. The detailed information on the related networks is given below.

- *foot-ball*(FOBA) (http://vlado.fmf.uni-lj.si/pub/networks/data/sport/football.htm accessed on 16 June 2020): The network describes the 22 soccer teams from 35 countries that participated in the World Championship in Paris, in 1998.
- Stmarks(STMA) (https://networkrepository.com/eco.php accessed on 16 June 2020): The St. Marks River (Florida) Flow network.
- Japanesemacaques(JAMA) (http://konect.cc/networks/moreno_mac/ accessed on 16 June 2020): This network contains dominance behavior in a colony of 62 adult female Japanese macaques.
- FWEW(FWEW): (https://networkrepository.com/eco.php accessed on 16 June 2020): The network of the food web in Everglades Graminoids.
- Lesmis(LESM) (http://konect.cc/networks/moreno_lesmis/ accessed on 16 June 2020): The network consists of the characters in Victor Hugo’s novel “Les Miserables”.
- FWMW(FWMW) (https://networkrepository.com/eco.php accessed on 16 June 2020): The network of the food web in Mangrove Estuary.
- Meetings(MEET) (https://zenodo.org/record/3938818 accessed on 16 June 2020): The Meetings network is built by analyzing the judicial documents of an anti-mafia operation called Montagna.
- FWFW(FWFW) (https://networkrepository.com/eco.php accessed on 16 June 2020): The network of the food web in Florida Bay.
- email167(EMAI) (https://networkrepository.com/ia-radoslaw-email.php accessed on 16 June 2020): The internal email communication network between employees of a mid-sized manufacturing company.
- Residencehall(REHA) (http://konect.cc/networks/moreno_oz/ accessed on 16 June 2020): This network contains friendship ratings between 217 residents living at a residence hall located on the Australian National University campus.
- Celegans(CELE) (http://konect.cc/networks/dimacs10-celegansneural/ accessed on 16 June 2020): The neural network of Caenorhabditis elegans.
- USAir97(USAI) (http://vlado.fmf.uni-lj.si/pub/networks/data/mix/USAir97.net accessed on 16 June 2020): The network consists of the US air transportation system.

The topological statistical characteristics of these real-world weighted graph data are listed in Table 3, where each row from left to right is the network name, number of nodes *n*, number of edges *m*, average degree <*k*>, average strength <*s*>, average clustering coefficient <*c*>, average weighted clustering coefficient $<\;\;c_{w}\;\;>$ and graph density ρ, respectively. Note that the self-connections and multiple edges in these weighted graph data are removed before calculating their topological statistical characteristics.

### 4.2. Benchmark Algorithms

Here, we introduce several similarity measures that are usually used for experimental comparison in the most similar node mining and link prediction. The basic motivation and definition of these similarity measures are given below.

The ***CN*** index directly regards the number of all common neighbors between two nodes as their similarity, which is

(13) $$S_{xy}^{CN}=\left|N\left(v_{x}\right)\cap N\left(v_{y}\right)\right|,$$

where $N\left(v_{x}\right)\cap N\left(v_{y}\right)$ represents the common neighbor set of $v_{x}$ and $v_{y}$, $N(v_{x})$ is the set of neighbors of $v_{x}$, and the |*V*| denotes the cardinality of set *V*.

The ***WCN*** index is the weighted version of *CN* index, which is defined as

(14) $$S_{xy}^{WCN}=\sum_{v_{z}\in N\left(v_{x}\right)\cap N\left(v_{y}\right)}w_{xz}+w_{yz},$$

where $w_{xz}=w_{zx}$ expresses the weight of the edge connecting $v_{x}$ and $v_{z}$.

The ***AA*** index is the extended version of the *CN* index, whose advantage is to refine the simple count of common neighbors. For the *AA* index, it gives less weight to the common neighbors with a greater degree, which is defined as

(15) $$S_{xy}^{AA}=\sum_{v_{z}\in N\left(v_{x}\right)\cap N\left(v_{y}\right)}\frac{1}{logk_{z}}.$$

The ***WAA*** index is the weighted version of *AA* index, which is defined as

(16) $$S_{xy}^{WAA}=\sum_{v_{z}\in N\left(v_{x}\right)\cap N\left(v_{y}\right)}\frac{w_{xz}+w_{yz}}{log(1+s_{z})}.$$

where $s_{z}$ may be smaller than 1, so we use $log(1+s_{z})$ in the above equation to avoid a negative score.

The ***LRW*** index is a similarity measure based on the local random walk of particles between two nodes, and its calculation expression is

(17) $$S_{xy}^{LRW}=\frac{k_{x}}{2\cdot |E|}\cdot \pi_{xy}\left(t\right)+\frac{k_{y}}{2\cdot |E|}\cdot \pi_{yx}\left(t\right).$$

where |E| is the number of the edges in graph data, and $\pi_{xy}\left(t\right)$ is obtained according to the density vector evolution equation: $\vec{\pi_{x}}\left(t+1\right)=P^{T}\cdot \vec{\pi_{x}}\left(t\right)$. In the density vector evolution equation, the *P* is the transition probability matrix, *T* is the matrix transpose, and *t* > 0 is the number of steps the particle takes to walk between two nodes. In this paper, *t* is specified as 3.

The ***RE-LRW*** index is a similarity measure of the local random walk based on the relative entropy. In the definition of the *RE-LRW* index, the probability distribution $P(v_{x})$ of $v_{x}$ is constructed in terms of the transition probability that it reaches other nodes after a three-step walk. Then, according to the degree centrality of each node, the transition probability of the top *d* important nodes is selected to form the *d*-dimensional probability distribution. Finally, the relative entropy is used to measure the difference between the transition probability distributions of the top *d* important nodes and all nodes in the graph data.

The ***LRE*** index is proposed with the help of the local structure of each node and relative entropy. Thereinto, the local structure of each node can be represented by utilizing the degree distribution of each node. After that, the probability distribution of each node can be obtained by normalizing all elements in their degree distribution. Finally, the difference between the probability distributions of two nodes can be measured by employing relative entropy, and then their similarity can be calculated accordingly.

### 4.3. Evaluation Metrics

In experiment, the performance of all similarity measures in the most similar nodes mining and link prediction are tested. For this reason, some evaluation metrics need to be introduced. In the most similar node mining, **the ratio of mutual most similar nodes**(abbreviated as ***MS***) is used to quantify the effectiveness of all similarity measures. In the link prediction, the **area under the receiver operating characteristic curve**(abbreviated as ***AUC***) is employed to quantify the prediction performance of all similarity measures.

***MS*** can be interpreted as the that if the most similar node of $v_{x}$ is $v_{y}$, then the most similar node of $v_{y}$ has a higher probability is $v_{x}$. Therefore, if the most similar node of $v_{x}$ is $v_{y}$ and the most similar node of $v_{y}$ is $v_{x}$, then $v_{y}$ and $v_{x}$ are mutually the most similar. For example, in the small-scale graph data with 10 nodes, the most similar node of $v_{1}$ is $v_{2}$ and the most similar node of $v_{2}$ is $v_{1}$, but other nodes are not mutually similar. Then, the number of the mutually most similar nodes in this small-scale graph data is equal to 2 and the *MS* is 0.2. Thus, the calculation expression of *MS* is

(18) $$MS=\frac{n_{ms}}{n},$$

where $n_{ms}$ denotes the number of the mutually most similar nodes. In general, the better the performance of a similarity measure, the larger the *MS* value obtained.

***AUC*** can be interpreted as the probability that an edge randomly selected in the test set is assigned a higher similarity than an edge randomly selected in the unknown edge set. After *r* times independent comparisons, if there are $r_{1}$ times that the similarity of the test edge is greater than that of the unknown edge and $r_{2}$ times that they have the same similarity, then the *AUC* value can be calculated as

(19) $$AUC=\frac{2r_{1}+r_{2}}{2r},$$

where *r* = 10,000 indicates the number of times that carried out the comparison of similarity in this paper.

## 5. Results

In this section, the performance of the *REDD* index and seven benchmark indices in the most similar node mining and link prediction is evaluated by employing two evaluation metrics: *MS* and *AUC*. There may be some statistical errors in the prediction accuracy due to the training set and test set being randomly divided during the link prediction. For reducing these errors, the final prediction accuracy of each index is the average value of running 30 independent experiments in all graph data. Furthermore, the training set proportion is specified as 0.9 in performing the link prediction.

### 5.1. Analysis of MS Results

**Figure 1:**
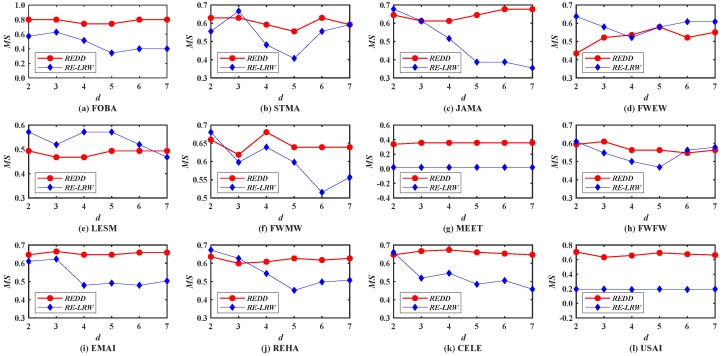
*MS* curves of *REDD* index and *RE-LRW* index with different dimensions *d*.

First of all, the performance of the *REDD* and *RE-LRW* indices are evaluated by using the *MS* metric. In Figure 1, we investigate the impact of different dimensions *d* on the *MS* values of the *REDD* index and *RE-LRW* index. From the results, one can observe that the *MS* curves of *RE-LRW* index have a large variation range in 10 out of 12 graph data. The *MS* curves of *RE-LRW* index are relatively flat in MEET and USAI, while its *MS* curves are considerably low. Moreover, it can be seen that the *MS* value of the *RE-LRW* index is almost close to 0 in MEET. This may be because there are some non-connected subgraphs in MEET, which will interrupt the random walk between nodes, resulting in the poor performance of the *RE-LRW* index. In contrast, the *REDD* index is not affected by the disconnection between nodes and can achieve good results in MEET. It can also be found that the *REDD* index can maintain high *MS* curves in most graph data, while keeping the variation of *MS* curves small. In particular, the *MS* curves of the *REDD* index are clearly higher than that of the *RE-LRW* index in FOBA, MEET, and USAI. From the above discussion, the *REDD* index owns a greater performance in the most similar node mining.

To compare the effectiveness between the *REDD* index and the seven benchmark indices, Table 4 lists the *MS* results of all indices in 12 weighted graph data. Note that the best *MS* value of each row is highlighted by using boldface. Furthermore, the *RE-*$LRW_{opt}$ and $REDD_{opt}$ are used to represent the *RE-LRW* and *REDD* indices with the optimal *MS* value in different dimensions *d*, respectively. From the results, it can be found that the *MS* values of the relative entropy-based indices are higher than those of the local structure-based indices and the random walk-based index. This indicates that it is indeed effective for the similarity measures based on relative entropy in the similarity calculation.

In these local structure-based indices, the *MS* values of *CN* and *WCN* indices have a greater difference in FOBA, MEET, FWFW, REHA, CELE, and USAI. For instance, the *MS* values of *CN* index are lower than those of the *WCN* index in FOBA and REHA, while the *MS* values of the *CN* index are higher than those of the *WCN* index in MEET, WFW, CELE, and USAI. This may be caused by the strong and weak ties in the weighted graph data. This phenomenon is also true for *AA* and *WAA* indices. Thus, it is necessary to comprehensively consider the degree and strength of nodes to avoid the influence of strong and weak ties.

Compared with the *RE-LRW* indices, the *LRW* index has lower *MS* values in most graph data, except MEET. Thus, there are many general similar nodes when the *LRW* index is used for the similarity calculation. At the same time, It reflects that the similarity measure based on the relative entropy can reduce the dependence on the large-degree nodes, and so the similarity of nodes can be better characterized.

For *LRE* and *REDD* indices, they can maintain higher *MS* values in 12 graph data. From the results, one can find that there are no general similar nodes when *LRE* and *REDD* indices are applied to calculate the similarity of nodes. Despite there being some non-connected subgraphs in MEET, the *LRE* and *REDD* indices still perform well. The *MS* values of the *LRE* and *REDD* indices are more than 0.4000, but the *MS* values of the *REDD* index can reach 0.7048 in USAI and 0.8000 in FOBA. Taken together, the *REDD* index has better performance during the most similar node mining.

### 5.2. Analysis of Scatter Diagram

In the most similar nodes mining, the scatterdiagrams are also used to validate the performance of the similarity measure. To make the experimental results distinguishable, the scatter diagrams of all indices are merely given in the graph data with more than 100 nodes. In scatter diagrams, the horizontal ordinate represents the label of nodes, and the vertical coordinates denote the label of the most similar nodes for the node in the horizontal ordinate. Therefore, the nodes should be scattered on the two-dimensional plane as much as possible in the scatter diagram. If the nodes are concentrated near the diagonal line or present a straight line (i.e., a large number of nodes are most similar to the same node), then the performance of this similarity measure is poor.

**Figure 2:**
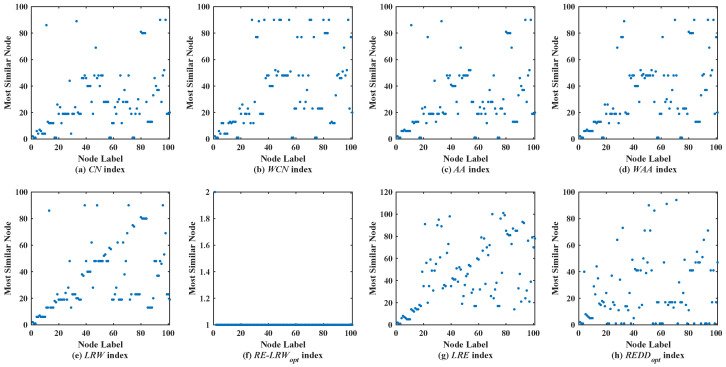
Scatter diagrams of the *REDD* index and seven benchmark indices in MMET.

Figure 2 shows the scatter diagrams of eight indices in MMET, where the degree of node $v_{19}$ is the largest, and the degree of node $v_{48}$ is the second largest. From the scatter diagrams of the *CN*, *WCN*, *AA*, *WAA*, and *LRW* indices, one can see that many nodes are similar to the nodes $v_{19}$ and $v_{48}$. It indicates that there are generally similar nodes in the measurement results of these indices. Additionally, there are no generally similar nodes in the measurement results of the *RE-LRW* index, but its scatter diagram has poor symmetry. From the scatter diagrams of the *LRE* and *REDD* indices, there are neither many nodes similar to the nodes of a large degree nor many nodes clustered on a straight line. Thus, the performance of *LRE* and *REDD* indices is outstanding in MEET.

**Figure 3:**
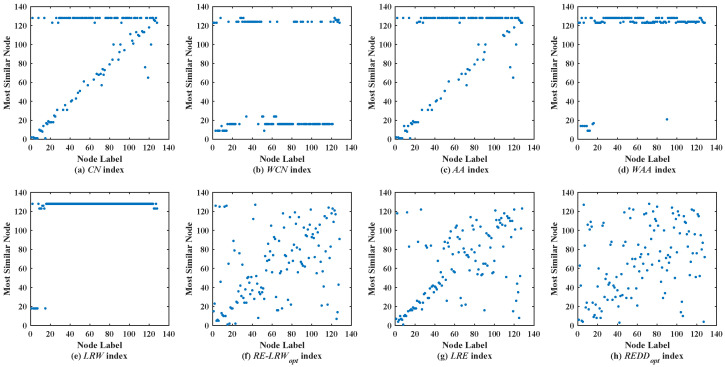
Scatter diagrams of the *REDD* index and seven benchmark indices in FWFW.

Figure 3 shows the scatter diagrams of eight indices in FWFW, where the degree of node $v_{128}$ is the largest, the degree of node $v_{123}$ is the second-largest. From the scatter diagrams of the *CN*, *WCN*, *AA*, *WAA*, and *LRW* indices, it can be seen that there are generally similar nodes $v_{128}$ and $v_{123}$. This leads to the scatter diagrams of these indices having poor symmetry. In contrast, the scatter diagrams of the *RE-LRW*, *LRE*, and *REDD* indices show good symmetry, while in the scatter diagram of the *LRE* index, one can also see that many nodes are concentrated near the diagonal line. It indicates that the performance of the *LRE* index is not as good as that of the *RE-LRW* and *REDD* indices. Although the *MS* value of the *RE-LRW* and *REDD* indices is equal, the scatter diagram of the former is not as well dispersed as the latter. On balance, the performance of the *REDD* index is more reasonable.

**Figure 4:**
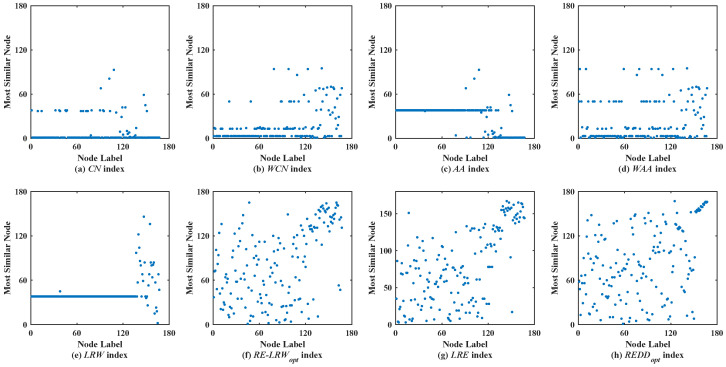
Scatter diagrams of the *REDD* index and seven benchmark indices in EMAI.

Figure 4 shows the scatter diagrams of eight indices in EMAI, where the degree of node $v_{38}$ is the largest, the degree of node $v_{37}$ is the second largest, and the degree of node $v_{45}$ is the third largest. Clearly, there are still generally similar nodes in the scatter diagrams of the *CN*, *WCN*, *AA*, *WAA*, and *LRW* indices. From the scatter diagrams of the *RE-LRW*, *LRE*, and *REDD* indices, one can find that these indices that use relative entropy can effectively distinguish the generally similar nodes. From the scatter diagrams of the three indices, it can be also seen that the *REDD* index performed better than the *RE-LRW* and *LRE* indices.

**Figure 5:**
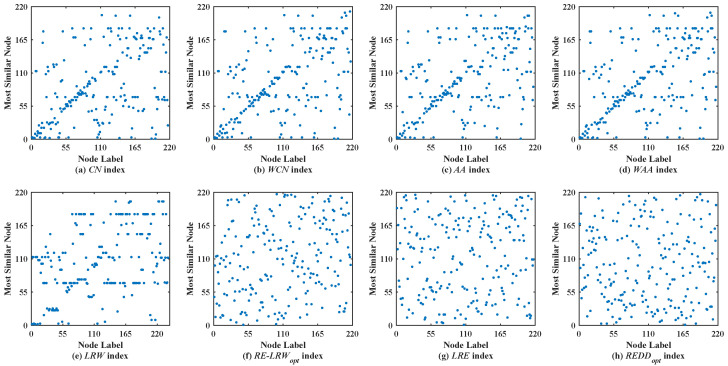
Scatter diagrams of the *REDD* index and seven benchmark indices in REHA.

Figure 5 shows the scatter diagrams of eight indices in REHA, where the degree of node $v_{70}$ is the largest, and the degree of node $v_{184}$ is the second largest. From the results, one can see that there are generally similar nodes $v_{70}$ and $v_{184}$ in the scatter diagrams of the *CN*, *WCN*, *AA*, *WAA*, and *LRW* indices. Moreover, many nodes are concentrated near the diagonal line in the scatter diagrams of the *CN*, *WCN*, *AA*, and *WAA* indices. Nevertheless, the scatter diagrams of the *RE-LRW*, *LRE*, and *REDD* indices still maintain a better symmetry. Despite the *MS* value of the *RE-LRW* index being higher than that of the *LRE* and *REDD* indices in REHA, the symmetry is better in the scatter diagrams of the *LRE* and *REDD* indices.

**Figure 6:**
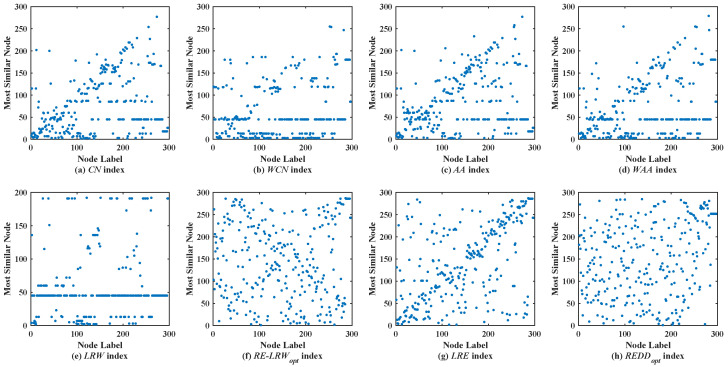
Scatter diagrams of the *REDD* index and seven benchmark indices in CELE.

Figure 6 shows the scatter diagrams of eight indices in CELE, where the degree of node $v_{45}$ is the largest, and the degree of node $v_{13}$ is the second largest. From the scatter diagrams of *CN*, *WCN*, *AA*, *WAA*, and *LRW* indices, it is not difficult to find that $v_{45}$ and $v_{13}$ become general similar nodes. Although there are not generally similar nodes in the scatter diagram of *LRE* index, many nodes are concentrated near the diagonal line. Thus, the performance of *LRE* index is superior to that of the *CN*, *WCN*, *AA*, *WAA*, and *LRW* indices, while the symmetry of the scatter diagram of *LRE* index is not as good as that of the scatter diagrams of *RE-LRW* and *REDD* indices. Furthermore, one can also find that the symmetry of the scatter diagram of *REDD* index is better than that of the scatter diagram of the *RE-LRW* index. On the whole, the *REDD* index does perform better in CELE.

**Figure 7:**
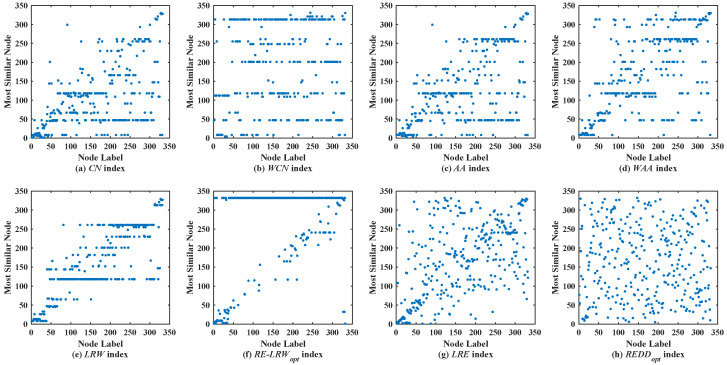
Scatter diagrams of the *REDD* index and seven benchmark indices in USAI.

Figure 7 shows the scatter diagrams of eight indices in USAI, where the degree of node $v_{118}$ is the largest, and the degree of node $v_{261}$ is the second largest. From the results in Figure 7, it can be observed that nodes $v_{118}$ and $v_{261}$ become generally similar nodes in the scatter diagrams of the *CN*, *WCN*, *AA*, *WAA*, and *LRW* indices.

From Figure 7, the *RE-LRW* index can really avoid the situation that the nodes of a large degree become generally similar nodes. Unfortunately, the most similar nodes of many nodes are clustered in a straight line in the scatter diagram of the *RE-LRW* index. It indicates that the symmetry of *RE-LRW* index still has great room for enhancement in USAI. In the scatter diagram of the *LRE* index, many nodes are rarely distributed near the diagonal line, so the *LRE* index has a relatively better symmetry. As for the *REDD* index, most of the nodes are not distributed near the diagonal line in its scatter diagram, and many large nodes do not become general similar nodes. Overall, the *REDD* index performs better in the USAI, compared to the other benchmark indices.

In this subsection, the performance of the *REDD* index and seven benchmark indices is analyzed. From the results of the *CN*, *WCN*, *AA*, and *WAA* indices, one can see that these only used the degree or strength of nodes are greatly affected by the strong and weak ties. Owing to the nodes of a large degree being more likely to be visited during the random walk, there are generally similar nodes in the measurement results of the *LRW* index. From the results of the *RE-LRW*, *LRE*, and *REDD* indices, it can be found that these indices use relative entropy and their own superior performance in the most similar nodes mining. Despite the results that the *RE-LRW* index is performed in MMET and USAI, it still has room for improvement. The *REDD* index comprehensively considers the degree and strength of nodes, while the *LRW* index only uses the degree of nodes. Hence, one can observe that the performance of the former is better than that of the latter from their results.

### 5.3. Analysis of Auc Results

**Figure 8:**
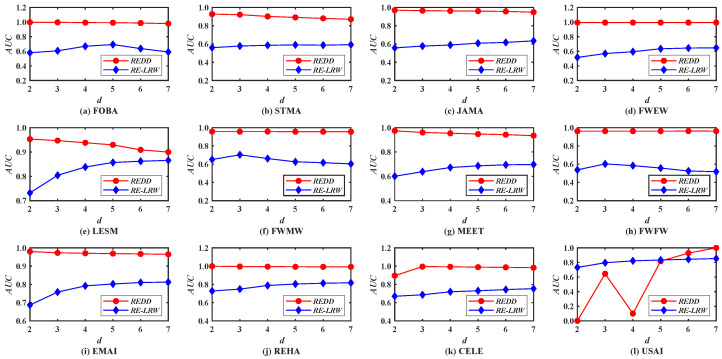
*AUC* curves of the *REDD* index and *RE-LRW* index with different dimensions *d*.

To test the effectiveness of similarity measure in link prediction, the *AUC* metric is further used to evaluate the performance of *REDD* index and seven benchmark indices. Figure 8 demonstrates the *AUC* curves of the *REDD* and *RE-LRW* indices when the dimension *d* changes from 2 to 7. From the results, one can observe that the variation amplitude of the *AUC* curves of the *RE-LRW* index are almost the same as that of the *REDD* index in the other graph data, except for USAI, while the accuracies of the *RE-LRW* index are far less than that of the *REDD* index under any dimension *d*. Despite the *AUC* values of *RE-LRW* index being higher than that of the *REDD* index when *d* is equal to 2, 3, 4, and 5 in USAI, the *AUC* values of the *REDD* index are clearly higher than that of the *RE-LRW* index when *d* is greater than 6. It indicates that the *REDD* index owns a greater potential during the link prediction. On the whole, compared with the *RE-LRW* index, the *REDD* index is more suitable for link prediction.

In the following, we analyze the effectiveness of the *REDD* index and seven benchmark indices during the link prediction. Table 5 lists the *AUC* results of all indices in 12 weighted graph data. From the results, one can observe that the *AUC* values of the *REDD* index are highest in 11 out of 12 graph data. Despite the *AUC* value of the *REDD* index being not as good as that of the four local indices in LESM, its *AUC* value is superior to that of the *LRW*, *RE-LRW*, and *LRE* indices.

From the results of the *CN*, *WCN*, *AA*, and *WAA* indices, one can also find that there is a great influence of the strong and weak ties on the similarity measure during the link prediction. For instance, the *AUC* values of the *WCN* and *WAA* indices are significantly greater than that of the *CN* and *AA* indices in FOBA, STAM, JAMA, FWEW, LESM, MEET, FWFW, EMAI, REHA, and CELE. This indicates that these similarity measures using the strength of nodes are easier to promote the formation of edges in these graph data, while the *AUC* values of the *WCN* and *WAA* indices are lower than that of the *CN* and *AA* indices in FWMW and USAI. It indicates that the fact of weak ties needs to be emphasized in the two graph data. Therefore, it may be more effective to construct the similarity measure by combining the degree and strength of nodes, such as the *REDD* index we designed.

From the results of the *LRW* and *RE-LRW* indices, although the *RE-LRW* index can enhance the performance of the *LRW* index in the most similar node mining, the effects of the *RE-LRW* index are inferior to those of the *LRW* index in link prediction. In other words, the *RE-LRW* index has a good performance in the most similar node mining, but its *AUC* results are quite poor. Therefore, the similarity measure considering only the degree of nodes might perform well only unilaterally in the most similar node mining or link prediction.

In a nutshell, the *REDD* index not only achieved good results in the most similar node mining, but also acquired a good application in link prediction. It further indicates that it is effective for comprehensively considering the role of the degree and strength of nodes to construct the similarity measure.

Generally, the low complexity is a vital factor in the design of an algorithm. In view of the complexity of local indices being relatively lower, we merely compare the running time of the *REDD*, *RE-LRW*, and *REDD* indices in 12 weighted graph data. Next, the time consumption of the *LRE*, *RE-LRW*, and *REDD* indices are compared by using the metric: *normalized time consumption* [33]. The *normalized time consumption* can be understood as the running time of index *a* in graph data *b* being normalized in the interval [0,1]. The corresponding calculation formula is ${\bar{t}}_{ab}=t_{ab}/\underset{a}{max}\left\{t_{ab}\right\}$, where $t_{ab}$ and ${\bar{t}}_{ab}$ are the running time and the normalized time consumption of index *a* in graph data *b*, respectively.

**Figure 9:**
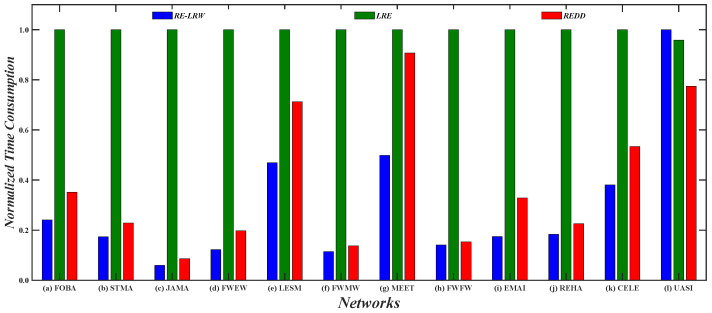
Normalized time consumption of three indices based on relative entropy.

Figure 9 shows the normalized time consumption of the *LRE*, *RE-LRW*, and *REDD* indices in 12 weighted graph data. From the result, the following three phenomena can be found. The *LRE* index runs the slowest in 11 out of 12 graph data. The time consumption of *RE-LRW* index increases with the increase in the number of nodes. The time consumption of the *REDD* index is at a medium level in LESM, MEET, and USAI. It is worth mentioning that the normalized time consumption of the *REDD* index is not the highest in all graph data. Hence, it is also feasible to apply the *REDD* index in large-scale weighted graph data if there is a better experimental environment. Above all, the *REDD* index owns a satisfactory time complexity in the process of link prediction.

### 5.4. Application to Simulated Networks

As described in the process of link prediction, many real-world graph data may be incomplete. Hence, it is difficult to design a similarity measure applicable to all real-world graphic data. To further verify the effectiveness of the *REDD* index, the *NW* small-world model is used to construct nine simulated graph data. Therefore, these simulated graph data are similar to real-world graph data. The *NW* model can establish the graph data with the different topological characteristics by adjusting the parameters *M* and *P*. For example, parameter *M* can be applied to adjust the average degree of the network, and parameter *P* can be utilized to regulate the average clustering coefficient of the network. The topological statistical characteristics of nine simulated networks are listed in Table 6. From Table 6, it can be observed that the node number of nine simulated graph data is specified as 100, and the topological statistical characteristics of these graph data are changed as the variation of parameters *M* and *P*.

**Figure 10:**
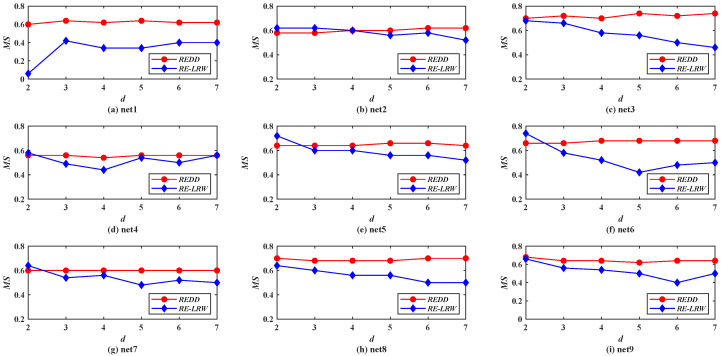
*MS* curves of *REDD* index and *RE-LRW* index in simulated networks.

**Figure 11:**
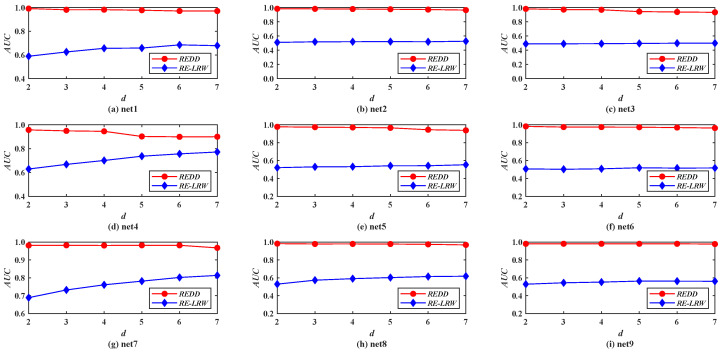
*AUC* curves of *REDD* index and *RE-LRW* index in simulated networks *d*.

**Figure 12:**
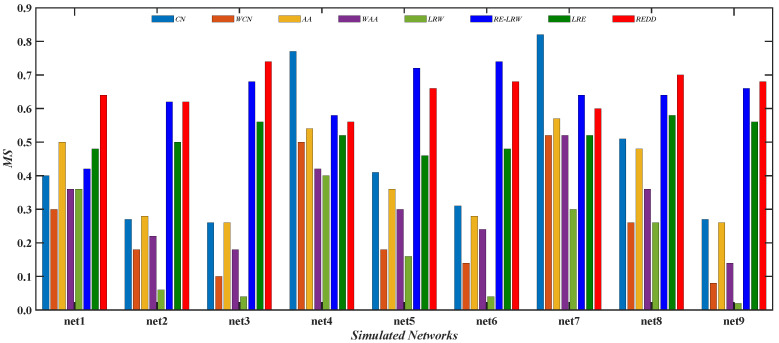
Comparison of *MS* values of *REDD* index and other indices in simulated networks.

**Figure 13:**
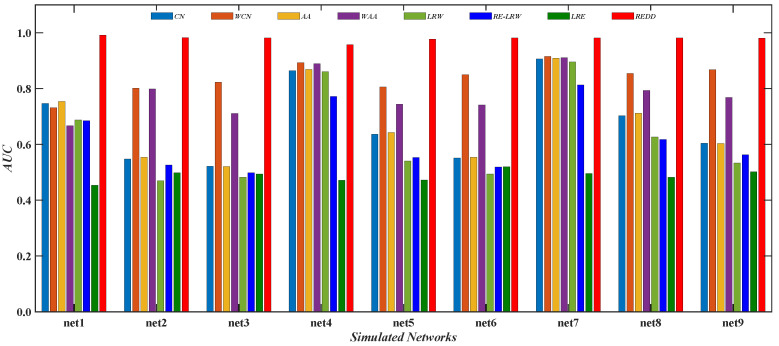
Comparison of *AUC* values of *REDD* index and other indices in simulated networks.

From the results of Figure 10, Figure 11, Figure 12 and Figure 13, it is not difficult find that the performance of the *REDD* index is hardly affected by the topological characteristics of graph data. Thus, in both the real-world graph data or in the simulated graph data, the *REDD* index has better performance in the most similar node mining and link prediction.

Figure 10 demonstrates the *MS* curves of the *REDD* and *RE-LRW* indices in nine simulated networks when the dimension *d* is changed from 2 to 7. Compared with the *MS* performance of the *REDD* and *RE-LRW* indices in real-world graph data, their *MS* performance shows higher accuracy in the simulated graph data. This indicates that the performance of the corresponding algorithm will be improved to some extent if the graph data can be collected more accurately. From Figure 10, one can observe that the *MS* curves of the *RE-LRW* index presents a large fluctuation range in different graph data. Thus, the robustness of the *RE-LRW* index still needs to be improved in simulated graph data.

Figure 11 shows the *AUC* curves of the *REDD* and *RE-LRW* indices in nine simulated networks when the dimension *d* is changed from 2 to 7. From the results in Figure 11, it can be observed that the *REDD* index can be better than the *RE-LRW* index in accuracy and robustness. Therefore, if the similarity measure based on relative entropy is proposed by only considering the degree of nodes, its performance may have no advantage in link prediction. Above all, these results in simulated graph data reflect that it is feasible to comprehensively take into account the degree and strength of nodes for enhancing the performance of the similarity measure based on the relative entropy once again.

Figure 12 gives the comparison of the *MS* values between the *REDD* index and seven benchmark indices in nine simulated networks. From the results, it can be seen that the performance of the *CN* and *AA* indices is better than that of their weighted version. It indicates that the *CN* and *AA* indices are more suitable for performing the most similar node mining in simulated networks. Moreover, it can be also found that the *MS* values of *CN* index is the highest in net4 and net7. This indicates that the *CN* index performs well in the graph data with a larger average clustering coefficient. Compared with the *RE-LRW* index, the performance of *LRW* index seems to be less than ideal in both most similar node mining cases.

Figure 13 reports the comparison of the*AUC* values between the *REDD* index and seven benchmark indices in nine simulated networks. From the results, it can be seen that the performance of the *CN* and *AA* indices may be inferior to that of their weighted version. It indicates that these indices that only consider the degree or strength of nodes are also influenced by strong and weak ties in the weighted simulated graph data. From the results, the performance of the *LRW* index may have a greater advantage than that of the *RE-LRW* index in link prediction. Thus, the performance of the *RE-LRW* and *LRW* indices need to be further improved in the most similar node mining and link prediction. In addition, it can be found that despite the *MS* performance of the *RE-LRW* index being almost the same as that of the *REDD* index, the *AUC* performance of the former is far less than that of the latter. This may be because the *REDD* index comprehensively utilizes the degree and strength of nodes, which results in the performance of algorithm being enhanced. From the above analysis and discussion, the *REDD* index can also achieve good results in simulated networks after considering the degree and strength of nodes.

### 5.5. Summarization and Discussion

In the previous subsections, we verified the performance of the *REDD* index in the most similar nodes mining and link prediction. The corresponding figures and tables show the experimental results of the *REDD* index and seven benchmark indices in 12 real-world weighted graph data and 9 simulated weighted graph data. From these results, we can obtain the following several summarizations and discussions.

- From the results in Figure 2 and Figure 8, it can be seen that the *MS* and *AUC* curves of the *RE-LRW* and *REDD* indices change with the variation in dimension *d*. From the variation range for the corresponding curves, one can find that the *REDD* index has greater applicability in the most similar node mining and link prediction. This also proves that the conjecture and motivation are feasible in this paper.
- From the results of Table 3 and Table 4, one can observe that the *REDD* index owns higher *MS* and *AUC* values than the seven benchmark indices. In particular, the *AUC* values of *REDD* index are more than 94% in 12 weighted graph data. This is because the degree and strength of nodes are considered in the *REDD* index at the same time. That makes the *REDD* index fully combine the ability of nodes to collect and diffuse information. Not only that, but the *REDD* index considers that the similarity between two nodes is inversely proportional to the distance between them. Thus, the probability between nodes is obtained by utilizing a constant 1 to subtract their normalized distance during the construction of the *REDD* index. This makes the *REDD* index better able to describe the fact of generating an edge between two nodes.
- According to the results from Figure 2, Figure 3, Figure 4, Figure 5, Figure 6 and Figure 7, we can find that the measurement results of the *CN*, *WCN*, *AA*, *WAA*, and *LRW* indices result in the situation that some nodes of a large degree become general similar nodes, while the relative entropy-based *RE-LRW*, *LRE*, and *REDD* indices better avoid the above-mentioned situation. Despite the scatter diagrams of the *RE-LRW* and *LRE* indices showing relatively better effects, they are not as good as the *REDD* index. This may be because the degree and strength of nodes are not fully considered in the definition of the *RE-LRW* and *LRE* indices.
- From the results in Figure 9, one can observe that the *REDD* index has a reasonable time cost, compared with *RE-LRW* and *LRE* indices. This indicates that the time consumption for measuring the difference between the probability distributions is less than the time consumption for measuring the difference between transition probability distributions and degree distributions.
- According to the results from Figure 10, Figure 11, Figure 12 and Figure 13, we can see that the *REDD* index has also better performance in most simulated networks, compared with seven benchmark indices. This indicates that the performance of the *REDD* index is hardly influenced by the type and structure of the network. It also proves that the *REDD* index has good universality in the most similar node mining and link prediction.
- In this paper, we introduce the relative entropy into weighted graph data and propose a similarity measure of nodes. The proposed measure is tested in multiple graph data, including real-world and simulated graph data. According to the experimental results, we guess that the performance of the *REDD* index can be also further analyzed by using some statistical methods. For instance, the Monte Carlo approach can be used to describe the reliability and limits of the *REDD* index.

## 6. Conclusions

To further enhance the performance for the similarity measure of nodes in weighted graph data, we designed the relative entropy of the distance distribution based similarity measure of nodes. Considering that the degree of nodes reflects their ability to diffuse information and the strength of nodes reflects their ability to collect information, thus the structural weights of nodes were defined by integrating their degree and strength. On this base, the structural weights-based distance between two nodes was calculated with the help of the Euclidean distance formula, and then the distance distribution of each node also was obtained. Because the relative entropy was used to measure the similarity of nodes in this paper, it is necessary to give the probability distribution of nodes. Hence, the probability distribution of nodes was defined by normalizing their distance distribution. To save time cost, the similarity of nodes was calculated by measuring the difference between the probability distributions of the top *d* important nodes and all nodes in graph data. In 12 real-world and 9 simulated weighted graph data, the performance of the proposed algorithm and 7 benchmark algorithms was compared by utilizing 2 evaluation metrics. A large number of theoretical derivation and experimental analyses demonstrated that the proposed similarity measure of nodes was more advantageous in both most similar node mining and link prediction.

In a large amount of graph data with complex structures, the status of many nodes may be disturbed, as discussed in Ref. [34] on the problem of graph node perturbation. Therefore, the influence of graph node perturbation will be considered in our algorithm framework to further validate the effectiveness of the proposed algorithm in future work.

## Figures and Tables

**Table 1:** Notations and descriptions.

Notations	Descriptions
$k_{x}$	The degree of node $v_{x}$, i.e., $k_{x}=\sum_{y=1}^{n}a_{xy}$ is the sum of the number of edges connected to node $v_{x}$
$s_{x}$	The strength of node $v_{x}$, i.e., $s_{x}=\sum_{y=1}^{n}w_{xy}$ is the sum of weights on all edges connected to node $v_{x}$
$s_{xy}$	The similarity of node $v_{x}$ and node $v_{y}$

**Table 2:** Experimental environment.

Parameter	Parameter Value
RAM	16 GB
Speed	1.8 GHz
Programming	MATLAB 2020a
CPU	AMD Ryzen 7 4800U
System	Windows 10 system with 8 cores

**Table 3:** Topological statistical characteristic of the real-world weighted graph data.

Network	*n*	*m*	<*k*>	<*s*>	<*c*>	$<\;\;c_{w}\;\;>$	ρ
FOBA	35	118	6.7429	16.8571	0.3708	0.7721	0.1983
STMA	54	350	12.9630	76.2991	0.4128	0.8310	0.2446
JAMA	62	1167	37.6452	78.5484	0.6671	1.2649	0.6171
FWEW	69	880	25.5072	578.2227	0.5521	0.1372	0.3751
LESM	77	254	6.5974	21.2987	0.7355	2.1892	0.0868
FWMW	97	1446	29.8144	98.9173	0.4683	0.4917	0.3106
MMET	101	256	5.0693	7.8416	0.7621	1.0667	0.0507
FWFW	128	2075	32.4219	54.0528	0.3364	0.2570	0.2540
EMAI	167	3250	38.9222	<*s*> ^1^	0.6864	$<\;\;c_{w}\;\;>$ ^2^	0.2345
REHA	217	1839	16.9493	83.2074	0.3628	1.8604	0.0785
CELE	297	2148	14.4646	59.3872	0.3079	1.4033	0.0489
USAI	332	2126	12.8072	0.9240	0.7494	0.0321	0.0387

^1^<s> = 1,264,299,248,759.5900; ^2^ $<\;\;c_{w}\;\;>$ = 9,386,734,484.3073.

**Table 4:** Comparison of the *MS* values between the *REDD* index and the other benchmark indices.

Network	*CN*	*WCN*	*AA*	*WAA*	*LRW*	*RE-* ${LRW}_{opt}$	*LRE*	${REDD}_{opt}$
FOBA	0.0875	0.1429	0.2286	0.2286	0.0571	0.6286	0.5143	**0.8000**
STMA	0.1111	0.0741	0.0741	0.0741	0.0370	**0.6667**	0.5556	0.6296
JAMA	0.0323	0.0323	0.0323	0.0323	0.0323	**0.6774**	0.5484	**0.6774**
FWEW	0.0870	0.0870	0.0870	0.0580	0.0290	**0.6377**	0.5797	0.5797
LESM	0.0779	0.0909	0.0779	0.0779	0.0260	**0.5714**	0.4675	0.4935
FWMW	0.0722	0.0412	0.0619	0.0206	0.0206	**0.6804**	0.5567	**0.6804**
MEET	0.1584	0.0990	0.1782	0.1386	0.1980	0.0198	**0.5941**	0.3564
FWFW	0.1094	0.0469	0.0781	0.0156	0.0156	**0.6094**	0.4688	**0.6094**
EMAI	0.0120	0.0120	0.0120	0.0120	0.0120	0.6108	0.5389	**0.6647**
REHA	0.1705	0.2028	0.1751	0.2080	0.0645	**0.6728**	0.4977	0.6359
CELE	0.1919	0.0572	0.1886	0.1145	0.0135	0.6599	0.5455	**0.6734**
USAI	0.0482	0.0120	0.0783	0.0602	0.0301	0.1958	0.5542	**0.7048**

**Table 5:** Comparison of the *AUC* values between the *REDD* index and the other benchmark indices.

Network	*CN*	*WCN*	*AA*	*WAA*	*LRW*	*RE-* ${LRW}_{opt}$	*LRE*	${REDD}_{opt}$
FOBA	0.6811	0.7876	0.6788	0.7626	0.8158	0.6925	0.5484	**0.9927**
STMA	0.6487	0.7007	0.6671	0.7204	0.8028	0.5925	0.4875	**0.9328**
JAMA	0.6789	0.9696	0.6794	0.9092	0.6635	0.6346	0.5196	**0.9750**
FWEW	0.6871	0.7039	0.6937	0.7075	0.8803	0.6477	0.5471	**0.9967**
LESM	0.9549	**0.9767**	0.9638	0.9763	0.9334	0.8656	0.7356	0.9489
FWMW	0.7094	0.6374	0.7103	0.6973	0.8565	0.7031	0.6429	**0.9577**
MEET	0.9585	0.9692	0.9692	0.9704	0.9185	0.6975	0.5960	**0.9757**
FWFW	0.6108	0.6302	0.6124	0.6504	0.8616	0.6031	0.5331	**0.9627**
EMAI	0.9206	0.9512	0.9216	0.9473	0.9252	0.8126	0.7594	**0.9808**
REHA	0.8959	0.9464	0.9022	0.9309	0.8761	0.8175	0.5615	**0.9991**
CELE	0.8498	0.8747	0.8654	0.8828	0.9014	0.7533	0.5587	**0.9944**
USAI	0.9546	0.9514	0.9671	0.9670	0.9617	0.8537	0.6665	**0.9832**

**Table 6:** Topological statistical characteristics of nine simulated networks.

Network	*M*	*P*	*n*	*m*	<*k*>	<*s*>	<*c*>	$<\;\;c_{w}\;\;>$	ρ
net1	2	0.0100	100	304	6.0800	503.1200	0.2762	19.5248	0.0614
net2	2	0.0500	100	680	13.6000	5463.8800	0.1590	62.7033	0.1374
net3	2	0.1000	100	1128	22.5600	24,220.1600	0.2333	253.2312	0.2279
net4	3	0.0100	100	374	7.4800	881.9200	0.4223	45.9759	0.0756
net5	3	0.0500	100	760	15.2000	7555.1600	0.2169	103.4878	0.1535
net6	3	0.1000	100	1172	23.4400	27,018.8800	0.2548	292.4191	0.2368
net7	4	0.0100	100	483	9.6600	1877.0000	0.4747	86.6363	0.0976
net8	4	0.0500	100	828	16.5600	9526.5600	0.2682	147.5209	0.1673
net9	4	0.1000	100	1240	24.8000	32,116.8800	0.2801	360.9700	0.2505

## Data Availability

Not applicable.
